# Mineral Springs of the United States and Canada

**Published:** 1873-11

**Authors:** 


*The Mineral Springs of the United States and Canada, with Analyses and Notes on the Prominent Spas of Europe, and a list of Sea-Side Resorts.* By George E. Walton, M. D. New York: D. Appleton & Co., 1873. Buffalo: Martin Taylor.

This work which consists of some three hundred and ninty pages, considers a subject which is of some importance in medical science. The opinions of the medical profession concerning the uses of mineral waters in the treatment of diseases are varied; some consider that they have no special therapeutic value and look upon them with no favor, while others acknowledging at the same time that too much has been expected of these therapeutical agents, look upon them as possessing certain medicinal properties, which are not to be overlooked or slighted in the treatment of disease. Dr. Walton's book is an admirable *resume* of the subject in which he gives a brief account of the prominent springs of the United States and Canada their therapeutical indications and value. The locality of these springs is given, and various other directions which will be of value to the reader. Physicians are often called to advise in the selection of a Mineral Spring, and this work will aid them very materially in the task.

